# A nationwide, multi‐institutional collaborative retrospective study of colorectal neuroendocrine tumors in Japan

**DOI:** 10.1002/ags3.12403

**Published:** 2020-11-17

**Authors:** Tatsuro Yamaguchi, Keiichi Takahashi, Kazutaka Yamada, Hiroyuki Bando, Hideo Baba, Masaaki Ito, Kimihiko Funahashi, Hideki Ueno, Shin Fujita, Seiji Hasegawa, Yoshiharu Sakai, Kenichi Sugihara

**Affiliations:** ^1^ Department of Surgery Tokyo Metropolitan Cancer and Infectious Diseases Center Komagome Hospital Tokyo Japan; ^2^ Department of Surgery Coloproctology Center Takano Hospital Kumamoto Japan; ^3^ Department of Gastroenterological Surgery Ishikawa Prefectural Central Hospital Kanazawa Japan; ^4^ Department of Gastroenterological Surgery Graduate School of Life Sciences Kumamoto University Kumamoto Japan; ^5^ Department of Colorectal Surgery National Cancer Center East Kashiwa Japan; ^6^ Department of General and Gastroenterological Surgery Toho University Omori Medical Center Tokyo Japan; ^7^ Department of Surgery National Defense Medical College Tokorozawa Japan; ^8^ Department of Surgery Tochigi Cancer Center Utsunomiya Japan; ^9^ Saiseikai Yokohama‐shi Nanbu Hospital Yokohama Japan; ^10^ Department of Surgery Kyoto University Kyoto Japan; ^11^ Tokyo Medical and Dental University Tokyo Japan

**Keywords:** neuroendocrine neoplasms, neuroendocrine tumors, rectal tumors

## Abstract

**Aim:**

Neuroendocrine tumors (NETs) are one of the subtypes of neuroendocrine neoplasms and are defined as epithelial neoplasms with predominant neuroendocrine differentiation. The aim of this study was to clarify the clinicopathological characteristics of colorectal NETs through a nationwide retrospective study in Japan.

**Methods:**

This multicenter retrospective cohort study of NETs in Japan was conducted by the study group of the Japanese Society for Cancer of the Colon and Rectum. In this study, we aimed to clarify the characteristics of Japanese patients with colorectal NETs. This cohort study included patients with colorectal NETs who were treated from January 2011 to December 2015.

**Results:**

Most NETs developed in the lower rectum. Predictive factors of lymph node metastasis included size (>10 mm), depth of invasion (muscular propria or greater), NET grade (NET G2), depressed lesion of the tumor, and lymphovascular infiltration. In particular, depressed lesion of the tumor and lymphovascular infiltration were independent predictive factors of lymph node metastasis. The presence of an increased number of these predictive factors increased the lymph node metastasis rate.

**Conclusion:**

Surgical resection with lymph node dissection is considered in the colorectal NETs patients with predictive factors of lymph node metastasis, the number of which is correlated with incidence of lymph node metastasis.

## Graphical abstract

This Japanese multicenter retrospective study revealed clinicopathological characteristics and the risk factors of lymph node metastasis in colorectal neuroendocrine tumors.

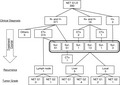

## 1 INTRODUCTION

Neuroendocrine tumors (NETs) are one of the subtypes of neuroendocrine neoplasms (NENs) and are defined as epithelial neoplasms with predominant neuroendocrine differentiation. Since neuroendocrine cells are distributed widely throughout the body, and NENs can arise at various locations, including the respiratory system and the digestive system.^1^ The World Health Organization (WHO) previously proposed a classification scheme for digestive NENs that divides them into three categories based on the mitosis count and Ki‐67 labeling index value: NET G1, NET G2, and neuroendocrine carcinoma (NEC).^2^ In particular, a mitotic count of <2 per 10 high‐power fields (HPFs) and/or a Ki‐67 index <3% corresponds to NET G1, a mitotic count of 2‐20 per 10 HPFs and/or a Ki‐67 index of 3%‐20% corresponds to NET G2, and a mitotic count of >20 per 10 HPFs and/or a Ki‐67 index >20% corresponds to NEC. In 2019, the WHO revised its former classification scheme and instead established a well‐differentiated subtype as NET G3 from among those cases previously classified as NEC.^3^

A total of five to seven new digestive NET cases per 100 000 people per year are diagnosed,^4^, ^5^ and colorectal NETs, including appendiceal NETs, account for approximately 50% of all digestive NETs.^4^, ^6^ Difference in the location of development of colorectal NETs in white and non‐white patients due to ethnic differences has been reported. It has been reported that NETs derived from the hindgut were predominant in non‐white patients, whereas those derived from the midgut were predominant in white patients.^4^, ^6^, ^7^, ^8^, ^9^

To date, only few reports have analyzed a large number of colorectal NET cases. Thus, the aim of this study was to clarify the clinicopathological characteristics of colorectal NETs through a nationwide retrospective study in Japan.

## 2 METHODS

This multicenter retrospective cohort study of NETs in Japan was conducted by the study group of the Japanese Society for Cancer of the Colon and Rectum (JSCCR). In this study, we aimed to clarify the characteristics of Japanese patients with colorectal NETs. This cohort study included patients with colorectal NETs who were treated from January 2011 to December 2015. The NET classification was performed according to the 2010 edition of the WHO classification scheme. The cohort study protocol was approved by the JSCCR Ethics Committee and the institutional review board of each involved center. Clinical information was collected either from medical records or directly from the patients.

The data in this study are presented as totals, medians (ranges or standard deviations), or percentages (95% confidence intervals). Statistical analysis was performed using Fisher's exact test and the Mann‐Whitney *U* test. Multivariable logistic regression models were performed to identify clinical and pathologic differences between lymph node‐negative and lymph node‐positive patients. We included the following variables in the multivariate logistic regression analysis: tumor size (≥10 mm or <10 mm), depressed lesion of the tumor, lymphovascular infiltration, invasion to muscular propria, and NET grade (G1 or G2), as these factors are well associated with lymph node metastasis in past reports.^10^, ^11^, ^12^, ^13^ The depressed lesions of the tumor were diagnosed endoscopically and/or histologically.

Statistical significance was defined at the level of *P* < .05. All statistical analyses were performed with EZR (http://www.jichi.ac.jp/saitama‐sct/SaitamaHP.files/statmedEN.html; Kanda, 2014; Saitama Medical Center, Jichi Medical University), which is a graphical user interface for R (version 3.6.0; The R Foundation for Statistical Computing). More specifically, the interface is a modified version of R Commander (version 2.5‐3) that was designed to add statistical functions frequently used in biostatistics.

## 3 RESULTS

**FIGURE 1:**
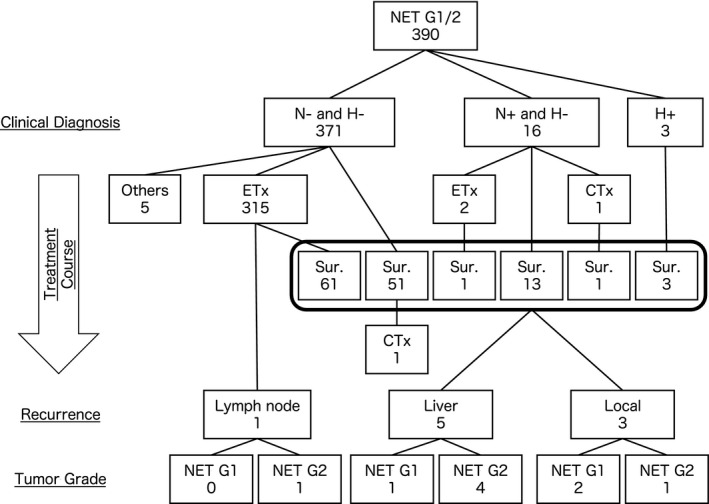
The treatment outline of colorectal neuroendocrine neoplasms patients. Clinical diagnosis, treatment course, recurrence site and tumor grade were shown. After a total of 390 patients had undergone surgical resection, liver metastasis in five patients and local recurrence in three patients occurred. CTx, chemotherapy; ETx, endoscopic treatment; H−, negative of liver metastasis; H+, positive of liver metastasis; N−, negative of lymph node metastasis; N+, positive of lymph node metastasis; NET, neuroendocrine tumor; Sur., surgical resection

In total, 416 patients from 25 institutions were enrolled in the present multicenter retrospective cohort study of colorectal NETs in Japan. Twenty‐six patients were excluded from the primary analysis because they could not be confirmed as colorectal NET cases. Thus, a total of 390 patients were included for the primary analysis (Figure 1).

The baseline characteristics of the patients are summarized in Table 1, and the number of patients categorized, based on NET grade, as NET G1 and NET G2 was 358 and 32, respectively. Based on the locations of NET development, the lower rectum was the most frequent site, followed by the upper rectum and then the rectosigmoid colon, while the appendix was involved in only 1.0% of all colorectal NETs. With respect to the depth of invasion, 95.8% of all colorectal NETs were limited to the submucosa layer. Finally, the frequency of lymph node and liver metastasis was 14.6% and 0.8%, respectively.

**TABLE 1:** Clinicopathological characteristics of colorectal neuroendocrine tumors

		All	NET G1	NET G2	*P* value
Age			59.0 (17‐86)	63.5 (38‐79)	.20
Gender	Male:female	238:152 (39.0%)	213:145 (40.5%)	25:7 (21.9%)	.039
Location	Upper rectum or more oral side:lower rectum	54:334 (86.1%)	45:311 (87.4%)	9:23 (71.9%)	.028
Size (mm)	<10:10≤	300:89 (22.9%)	288:70 (19.6%)	12:19 (61.3%)	<.0001
Depressed lesion	Negative:positive	273:71 (20.6%)	258:56 (17.8%)	15:15 (50.0%)	.0002
Liver metastasis	Negative:positive	387:3 (0.8%)	357:1 (0.3%)	30:2 (6.3%)	.019
Depth of invasion	pT1:pT2≤	365:16 (4.2%)	341:9 (2.6%)	24:7 (22.6%)	<.0001
Lymph node metastasis	Negative:positive	317:54 (14.6%)	301:40 (11.7%)	16:14 (46.7%)	<.0001
Lymphatic infiltration	Negative:positive	306:53 (14.8%)	289:41 (12.4%)	17:12 (41.4%)	.0002
Venous infiltration	Negative:positive	280:78 (21.8%)	269:61 (18.5%)	11:17 (60.7%)	<.0001
Chromogranin A	Negative:positive	82:218 (72.7%)	75:197 (72.4%)	7:21 (75.0%)	1
Synaptophysin	Negative:positive	22:271 (92.5%)	21:245 (92.1%)	2:26 (92.9%)	1

Abbreviations: G, grade; NET, neuroendocrine tumor.

The patients with colorectal NETs without any metastasis underwent endoscopic resection (n = 315) and surgical resection (n = 51). Of the 254 patients with colorectal NETs who received no additional treatment after endoscopic resection, one experienced lymph node recurrence. Additionally, after surgical resection, five patients experienced liver metastasis and three experienced local recurrence.

**FIGURE 2:**
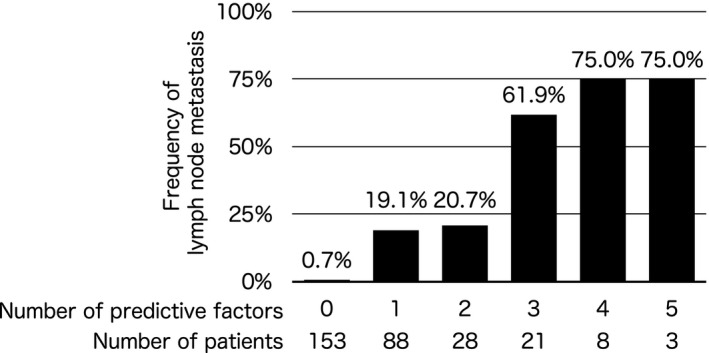
Frequency of lymph node metastasis according to number of predictive factors. There was significant correlation between frequency of lymph node metastasis and number of predictive factors, including size (>10 mm), depth of invasion (muscular propria or greater), NET grade (NET G2), depressed lesion of the tumor, and lymphovascular infiltration

The univariate analysis demonstrated that size (≥10 mm), depth of invasion (muscular propria or greater), NET grade (NET G2), depressed lesion of the tumor, and lymphovascular infiltration were predictive factors of lymph node metastasis (Table 2). Overall, the greater the number of predictive factors for lymph node metastasis, the greater the rate of lymph node metastasis (*P* < .0001; Figure 2). In addition, a multivariate analysis demonstrated that depressed lesion of the tumor and lymphovascular infiltration were independent predictive factors of lymph node metastasis.

**TABLE 2:** Univariate and multivariate analysis of the risk factors associated with lymph node metastasis

		Univariate analysis			Multivariate analysis	
		pN0	pN+	*P* value	Odds ratio	*P* value
Age		55.9 ± 13.7	58.3 ± 12.2	.36		
Gender	Male:female	190:127 (40.1%)	36:18 (33.3%)	.37		
Location	Upper rectum or more oral side:lower rectum	44:272 (86.1%)	8:46 (85.2%)	.83		
Size (mm)	<10:10≤	266:51 (16.1%)	23:31 (57.4%)	<.0001	0.54 (0.21‐1.43)	.22
Depressed lesion	Negative:positive	242:39 (13.9%)	20:28 (58.3%)	<.0001	4.98 (1.97‐12.60)	.0007
Tumor grade	G1:G2	301:16 (5.0%)	40:14 (25.9%)	<.0001	0.60 (0.193‐1.89)	.38
Liver metastasis	Negative:positive	316:1 (0.3%)	52:2 (3.7%)	.057		
Depth of invasion	pT1:pT2≤	303:9 (2.9%)	48:6 (11.1%)	.014	0.77 (0.15‐4.09)	.76
Lymphatic infiltration	Negative:positive	265:28 (9.6%)	28:24 (46.2%)	<.0001		
Venous infiltration	Negative:positive	253:40 (13.7%)	17:35 (67.3%)	<.0001		
Lymphovascular infiltration	Negative:positive	235:58 (19.8%)	42:10 (19.2%)	<.0001	15.1 (6.22‐36.90)	<.0001

Abbreviations: G, grade; pN0, negative lymph node metastasis; pN+, positive lymph node metastasis.

The clinicopathological characteristics of colorectal NETs according to liver metastasis were shown in Table 3. However, we performed neither a univariate nor a multivariate analysis because of the small number of the patients with liver metastasis.

**TABLE 3:** Clinicopathological characteristics of colorectal NET with/without liver metastasis

		Liver metastasis−	Liver metastasis+
Age		56.6 ± 13.4	44.3 ± 11.0
Gender	Male:female	233:151 (39.3%)	3:0
Location	Upper rectum or more oral side:lower rectum	53:329 (86.1%)	0:3
Size (mm)	<10:10≤	298:85 (22.2%)	1:2
Depressed lesion	Negative:positive	270:68 (20.1%)	1:2
Tumor grade	G1:G2	356:28 (7.3%)	1:2
Lymph node metastasis	Negative:positive	315:50 (13.7%)	1:2
Depth of invasion	pT1:pT2≤	361:14 (3.7%)	2:1
Lymphatic infiltration	Negative:positive	304:50 (14.1%)	0:2
Venous infiltration	Negative:positive	279:74 (21.0%)	0:2
Lymphovascular infiltration	Negative:positive	254:100 (28.2%)	0:2

Abbreviations: G, grade; NET, neuroendocrine tumor.

## 4 DISCUSSION

The present study demonstrated the following: (a) most NETs developed in the lower rectum; (b) predictive factors of lymph node metastasis included size (>10 mm), depth of invasion (muscular propria or greater), NET grade (NET G2), depressed lesion of the tumor, and lymphovascular infiltration, especially depressed lesion of the tumor and lymphovascular infiltration which were independent predictive factors of lymph node metastasis; (c) the presence of an increased number of these predictive factors increased the lymph node metastasis rate.

Among the colorectal NET cases, in this study of Japanese patients, the frequency of appendiceal NETs was only 1.0%, whereas the frequency of appendiceal NETs was reported to be approximately 10% in the United States.^12^ Since appendiceal NETs are also somewhat infrequent specifically among African Americans and Native Americans in the United States,^1^ environmental factors may not affect the development of appendiceal NETs.

Predicting lymph node metastasis by preoperative computed tomography is difficult.^14^ In general, it is recommended that the NET patients with some risk factors for lymph node metastasis undergo surgical treatment. It has previously been reported that tumor size (≥10 mm or <10 mm), depressed lesion of the tumor, lymphovascular infiltration, invasion into the muscular propria, and NET grade (G1 or G2) are predictive factors for lymph node metastasis.^10^, ^11^, ^12^, ^13^ These factors were associated with lymph node metastasis in this study as well. Additionally, we demonstrated that an increase in the number of these predictive factors increased the lymph node metastasis rate. Therefore, patients without any risk factors may not need to undergo surgical resection, as their lymph node metastasis rate is <1%.

On the other hand, several groups reported that rectal NETs patient with lymphovascular infiltration underwent only endoscopic resection and did not subsequently metastasize or recur.^15^, ^16^, ^17^ Recent systematic review reported that rectal NETs patients with lymphovascular infiltration were favorable, with only 0.3% of recurrence in the 1022 patients, and concluded that completion of radical surgery would not be absolutely necessary for LVI‐positive small rectal NETs treated with endoscopic resection.^18^ However, they also added that long‐term follow‐up of 10‐20 years is recommended to assess for any delayed recurrence in rectal NETs patients without surgical resection.

The liver is the second most common distant organ of metastatic NET following lymph node.^19^ Tumor size was reported to correlate with liver metastasis in rectal NET.^11^, ^19^, ^20^, ^21^, ^22^ Although rare in rectal NETs smaller than 1 cm, approximately 25% in those over 2 cm have liver metastasis. Few studies have reported the predictive factors of metachronous liver metastasis in colorectal NETs. We were also unable to perform either a univariate or multivariate analysis because of the small number of the patients who experienced metachronous liver metastasis. Larger studies are needed to elucidate the risk factor of liver metastasis in NET patients.

In conclusion, surgical resection with lymph node dissection should be considered in the colorectal NETs patients with predictive factors of lymph node metastasis, the number of which is correlated with incidence of lymph node metastasis. Our data will help in the decision‐making process for surgical resection with lymph node dissection in colorectal NETs patients.

The present study has some limitations that should be highlighted. First, although 390 patients with colorectal NET was a relatively large cohort for this field, the sample size was still too small for any definitive conclusions to be made; therefore, our findings need to be confirmed by an even larger study. Second, the observation period was short, as the study period ranged from January 2011 to December 2015. Therefore, we might have missed patients who might have experienced recurrence since December 2015. Nonetheless, considering that we elucidated features of colorectal NETs in Japanese patients in our investigation, we believe that our findings will help researchers and physicians alike to clarify colorectal NETs.

## DISCLOSURE

Funding: The present study was supported in part by the Japanese Society for Cancer of the Colon and Rectum.

Conflict of Interest: None declared.
